# Construction Notes

**Published:** 1893-07-08

**Authors:** 


### CONSTRUCTION NOTES.

BROMLEY AND BECKENHAM JOINT HOSPITAL. — The Board of the Bromley and Beckenham Joint Hospital have recommended the acceptance of Mr. Graty's tender for erecting the proposed alteration at the hospital for the estimated sum of £3,399, on the plans prepared by Mr. J. Ladds, architect, of Mecklenburg Square, London.

COTTAGE HOSPITAL AT THAMES DITTON.—The plans for the erection of a new cottage hospital at Thames Ditton have been approved by the Committee of Management, and a site has been obtained for the building. The contract for the building, at the estimated sum of £817, has been placed with Mr. W. Callingham, and during the past week the foundation has been got out. The site is in Weston Green Road, and the building, which is to be on two floors, is of pretty elevation with a west aspect. On the ground floor a passage runs through the building. On the south side is the day room and accident ward; on the north side the nurses' room, kitchen, and offices. On the first floor will be four bed-rooms, bathroom, and other conveniences. Behind the accident ward space is reserved for an extension when required. The architect is Mr. A. J. Style.

ENLARGEMENT OF WARRINGTON INFIRMARY.—About three years ago the increasing demand made upon the accommodation for patients at this infirmary induced the Committee to promote a scheme for enlarging the building. Plans were immediately drawn up and public subscriptions were invited. The first addition was the new dispensary. Then three additional wards were erected on the first floor and three others were erected on the top storey. Every accommodation has been placed in these rooms which is compatible with the latest and most approved of sanitation, the fireplaces, for instance, being situated in the centre of the room. Altogether these six wards will accommodate an additional 20 inmates; hitherto the total accommodation being for 23. Several alterations have been made to the operating-room, including the laying of fresh tiles on the floor; and also a waiting-room has been added on the ground floor, and an additional room for treating accidents.

NEW LAUNDRY AT ROYAL UNITED HOSPITAL, BATH.—This is the latest improvement to this hospital, and it is as compact and convenient as it is useful. A portion of the yard at the rear of the building has been excavated to the depth of several feet to accommodate the laundry, the leads of which are well below the windows on the ground floor of the institution. Five rooms opening one into the other comprise the department, which is easily reached from the basement. In the principal room two patent washing machines of latest design have been placed, also a powerful wringer, drying chambers, with several metal and wood troughs. The ironing-room is a bright and well-lighted apartment, provided with spacious benches, and a mangling machine. The floors are pitched with wooden blocks laid on concrete beds. A patent fan ventilator has been fixed. The machinery has been supplied by Messrs. Summersgales, of Keighley, the contractor being Mr. Webb, of Bath, and the designing architects, Messrs. Brown and Gill.

THE NEW SISTERS HOSPITAL, ST. ALBANS.—The freehold site of one acre for this infectious diseases hospital was selected and bought by Sir J. Blundell Maple, to whose munificent generosity St. Albans owes the existence of the new hospital. The need of such a building has been proved in a forcible manner during its erection by an outbreak of small-pox, which necessitated the Corporation erecting a temporary iron hospital for fourteen beds. This, however, is to remain as a useful adjunct to the now completed permanent hospital close by, the plans of which show three separate buildings connected by the covered ways, the hospital proper, the adminstrative block, and the outbuildings. In the adminstrative block is a large hall, a nurses' sitting-room, also two bed-rooms and a bath-room. Here, also, is the kitchen, with scullery, larder, and other offices. In this block is the caretaker's house, containing living-room, offices, and two bed-rooms. The hospital proper is a building 130 feet long, standing in the middle of the ground, comprising a centre of two floors, and side wings of one floor. The corridor, which runs the length of the building on the ground floor, is glazed in, but with ample ventilation, the windows being so constructed that in warm weather the sashes can be lifted entirely out. Entering the hospital on the north side, under the covered way from the adminstrative block, and crossing the last described corridor, one finds a spacious hall, in which are the stairs to the first floor wards, the linen store, hot water apparatus, and a nurse's room. Nurses' rooms and a ward are arranged alternately. There are four wards on the ground floor, and two, with another nurse's room between them, on the first floor, making six wards in all, and containing fourteen beds in all. The wards are so well isolated that they can each and all be used on necessity for either sex, or any disease. The floors are wax polished, all internal angles of the walls being rounded, and angles and corners for dust thus avoided. Detached from the rooms on either side of the corridor, are the closets and slop sinks; the earth closets being supplied by Messrs. Moule and Co. The outbuildings contain an ambulance shed, then a disinfecting chamber. The washhouse, fitted with every necessary machinery, is here, as also the drying closet, furnace and mangle, which with the cooking range and hot water apparatus have all been fixed by Messrs. Benham and Co., of London. A fuel store and a mortuary complete the outbuildings. The works have been carried out by Mr. C. Miskin, of St. Albans, under the superintendence of Mr. Morton. Mr. Glover is the architect. This munificent gift to the city is estimated to have cost Sir Blundell Maple £8,000.

